# A nested mechanistic sub-study into the effect of tranexamic acid versus placebo on intracranial haemorrhage and cerebral ischaemia in isolated traumatic brain injury: study protocol for a randomised controlled trial (CRASH-3 Trial Intracranial Bleeding Mechanistic Sub-Study [CRASH-3 IBMS])

**DOI:** 10.1186/s13063-017-2073-6

**Published:** 2017-07-17

**Authors:** Abda Mahmood, Ian Roberts, Haleema Shakur

**Affiliations:** Clinical Trials Unit, London School of Hygiene and Tropical Medicine, University of London, Keppel Street, London, WC1E 7HT UK

**Keywords:** Tranexamic acid, Intracranial haemorrhage, Cerebral ischaemia, Traumatic brain injury

## Abstract

**Background:**

Tranexamic acid prevents blood clots from breaking down and reduces bleeding. However, it is uncertain whether tranexamic acid is effective in traumatic brain injury. The CRASH-3 trial is a randomised controlled trial that will examine the effect of tranexamic acid (versus placebo) on death and disability in 13,000 patients with traumatic brain injury. The CRASH-3 trial hypothesizes that tranexamic acid will reduce intracranial haemorrhage, which will reduce the risk of death. Although it is possible that tranexamic acid will reduce intracranial bleeding, there is also a potential for harm. In particular, tranexamic acid may increase the risk of cerebral thrombosis and ischaemia. The protocol detailed here is for a mechanistic sub-study nested within the CRASH-3 trial. This mechanistic sub-study aims to examine the effect of tranexamic acid (versus placebo) on intracranial bleeding and cerebral ischaemia.

**Methods:**

The CRASH-3 Intracranial Bleeding Mechanistic Sub-Study (CRASH-3 IBMS) is nested within a prospective, double-blind, multi-centre, parallel-arm randomised trial called the CRASH-3 trial. The CRASH-3 IBMS will be conducted in a cohort of approximately 1000 isolated traumatic brain injury patients enrolled in the CRASH-3 trial. In the CRASH-3 IBMS, brain scans acquired before and after randomisation are examined, using validated methods, for evidence of intracranial bleeding and cerebral ischaemia. The primary outcome is the total volume of intracranial bleeding measured on computed tomography after randomisation, adjusting for baseline bleeding volume. Secondary outcomes include progression of intracranial haemorrhage (from pre- to post-randomisation scans), new intracranial haemorrhage (seen on post- but not pre-randomisation scans), intracranial haemorrhage following neurosurgery, and new focal ischaemic lesions (seen on post-but not pre-randomisation scans). A linear regression model will examine whether receipt of the trial treatment can predict haemorrhage volume. Bleeding volumes and new ischaemic lesions will be compared across treatment groups using relative risks and 95% confidence intervals.

**Discussion:**

The CRASH-3 IBMS will provide an insight into the mechanism of action of tranexamic acid in traumatic brain injury, as well as information about the risks and benefits. Evidence from this trial could inform the management of patients with traumatic brain injury.

**Trial registration:**

The CRASH-3 trial was prospectively registered and the CRASH-3 IBMS is an addition to the original protocol registered at the International Standard Randomised Controlled Trials registry (ISRCTN15088122) 19 July 2011, and ClinicalTrials.gov on 25 July 2011 (NCT01402882).

**Electronic supplementary material:**

The online version of this article (doi:10.1186/s13063-017-2073-6) contains supplementary material, which is available to authorized users.

## Background

### Traumatic brain injury (TBI) occurrence

TBI is a leading cause of death and disability worldwide. According to the World Health Organization, TBI will continue to be a major cause of death and disability by 2020 [1]. At least 200 per 100,000 people are killed or hospitalised each year after TBI [2], resulting in over 10 million deaths or hospitalisations each year [3]. TBI is the leading cause of death and disability in people below the age of 45 [4].

TBI patients can experience a loss in physical, behavioural or emotional functioning after the injury [5]. Severe TBI often results in motor impairment that persists for at least 3 years after the injury [6] and cognitive impairments are present for at least 6 months after injury [7]. Problems with memory following TBI significantly affect an individual’s quality of life [8]. Even with rehabilitation treatments, only 40–50% of TBI patients completely recover [9].

The increasing incidence of TBI can be explained by the rising frequency of traffic accidents in developing countries and rapidly motorising middle-income countries [10]. Projections of global mortality and burden of disease suggest that road traffic accidents will be the third major cause of death and disability by 2030, assuming a faster rate of socio-economic development [11]. Falls in older adults are the leading cause of TBI in high-income countries [12]. Given the global scope of this life threatening and potentially disabling condition, it is important to identify the most effective clinical care in this patient group.

### Intracranial haemorrhage occurrence

Intracranial bleeding is common after TBI and the larger the bleed the greater the risk of death and disability [13, 14]. In patients with mild TBI (Glasgow Coma Scale score ≥ 13), although bleeding can continue for up to 24 hours after injury, most bleeds stop progressing within a few hours of hospital admission [15]. Intracranial haemorrhage progression has been observed in half of moderate to severe head injury patients who had a median Glasgow Coma Scale score of 8 on admission and repeat computed tomography (CT) scans performed within 24 hours of injury [16, 17]. Patients who were scanned earlier after injury (≤3.5 hours vs. > 3.5 hours) were more likely to have expanding haematomas on CT performed 24 hours after injury (57% vs. 28%) [17]. If the initial CT scan was conducted more than 3.5 hours after injury, the percentage of patients with measurable changes in haematoma volume 24 hours after injury was reduced. In a subset of patients who had an intermediate scan (most of which were between 6 and 9 hours of injury), the mean volume change between the baseline and intermediate scan was 5.7 mL, whereas the difference in mean volume between the intermediate scan and the 24 hour scan was 0.03 mL [17]. Thus, the maximal change in intracranial haemorrhage volume occurs soon after injury.

A meta-analysis of 34 studies that reported the frequency of coagulopathy after TBI found that one third of patients with TBI have laboratory evidence of abnormal coagulation based on parameters such as fibrinogen, fibrin degradation products and antithrombin levels [18].

The risk of mortality in patients with coagulopathy after TBI is nine times higher than in TBI patients without coagulopathy (odds ratio (OR) 9.0, 95% confidence interval (CI) 7.3–11.6). The risk of unfavourable outcome as measured by the Glasgow Outcome Scale (score of 1–3) is more than 30 times higher in TBI patients with coagulopathy (OR 36.3, 95% CI 18.7–70.5) [18]. Decreased platelet counts, prolonged prothrombin time and partial thromboplastin time, and high levels of fibrinogen and D-dimer levels are observed in patients within the first 3 hours of TBI [19]. The highest D-dimer concentrations were found in the most severely injured patients [20], who have a higher risk of intracranial haemorrhage and mortality.

### Effectiveness of tranexamic acid in reducing haemorrhage

Tranexamic acid reduces bleeding by inhibiting the enzymatic breakdown of fibrin blood clots. Plasmin binds to fibrin via lysine-binding sites and then splits fibrin into fibrin degradation products. Tranexamic acid is a molecular analogue of lysine that inhibits fibrinolysis by reducing the binding of plasmin to fibrin.

A systematic review of 104 randomised trials of tranexamic acid in surgical patients found that it reduced the number of patients receiving a blood transfusion by one-third and halved the need for further surgery to control bleeding [21].

A large randomised trial of tranexamic acid treatment within an hour of acute traumatic injury found that it reduced the risk of death due to bleeding by about one-third (relative risk (RR) 0.68, 95% CI 0.57–0.82; *P* < 0.0001) [22, 23]. Treatment between 1 and 3 hours reduced the risk by about one-fifth (RR 0.79, 0.64–0.97; *P* = 0.03). There was no apparent increase in the risk of vascular occlusive events with tranexamic acid following acute trauma (RR 0.69, 95% CI 0.44–1.07; *P* = 0.096).

### Tranexamic acid as a potential treatment in TBI

Tranexamic acid is able to penetrate the blood–brain barrier and should be able to affect intracranial haemorrhage [24]. If tranexamic acid is effective following TBI, it should also be most effective when given soon after injury when intracranial bleeding is on-going [15]. Furthermore, if early increased fibrinolysis exacerbates bleeding and increases the risk of death [20], we would expect tranexamic acid to be most effective during this period.

However, there is also the potential for harm. In particular, tranexamic acid may increase the risk of cerebral thrombosis and ischaemia [25]. Cerebral ischaemia is an important secondary injury mechanism after TBI that worsens neurologic outcome and increases mortality [26, 27]. It can be precipitated by raised intracranial pressure, which can lead to cerebral hypo-perfusion [28–31]. In addition, thrombotic disseminated intravascular coagulation may increase the risk of cerebral micro-thrombi, which are often seen in the brains of TBI patients who die within 24 hours of injury [32]. By inhibiting fibrinolysis, tranexamic acid might increase the risk of cerebral ischaemia and thrombosis in TBI patients.

A systematic review identified two completed randomised trials of tranexamic acid in TBI patients [33]. The first randomised trial (*n* = 249) examined the effect of tranexamic acid in patients with extra-cranial bleeding but who also had TBI [34]. The second randomised trial (*n* = 229) examined the effect of tranexamic acid in patients with polytrauma and TBI or isolated TBI [35]. Both trials used information from pre- and post-randomisation CT scans to estimate the extent of bleeding and ischaemia. Both trials recruited patients who were within 8 hours of injury, yet they were not large enough to determine the balance of risks and benefits from tranexamic acid and whether this varies by time to treatment.

When the two randomised trials were combined in a meta-analysis, there was a statistically significant reduction in intracranial haemorrhage (RR 0.75, 95% CI 0.58–0.98; *P* = 0.03) and mortality (RR 0.63, 95% CI 0.40–0.99; *P* = 0.05) with tranexamic acid. In one trial, focal ischaemic lesions occurred in 5% of tranexamic acid-treated patients and 9% of placebo-treated patients (RR 0.51, 95% CI 0.20–1.32; *P* = 1.17) [34]. In the second trial, there were three strokes in the placebo group compared with none in the tranexamic acid group [35]. However, because the CIs for intracranial haemorrhage, death and ischaemic lesion outcomes are so wide, the quality of this evidence is low. Furthermore, the patients in the trials had extra-cranial bleeding in addition to intra-cranial bleeding. Because tranexamic acid reduces mortality in extra-cranial bleeding (CRASH-2), the mortality reduction seen in this trial could be from the extra-cranial injury rather than any effect on the brain injury. The effect of tranexamic acid on intracranial haemorrhage and thrombotic adverse effects, including stroke, remains uncertain.

There are three ongoing randomised trials of tranexamic acid versus placebo in patients with isolated TBI (NCT02645552, NCT01990768, NCT01402882). These will evaluate the effect of tranexamic acid on death, disability, vascular occlusive events and other adverse events in TBI. The ongoing trials will inform whether tranexamic acid can be given to those with TBI. To date, the CRASH-3 trial, with a planned sample size of 13,000 patients, will be the largest randomised trial into the effect of tranexamic acid in TBI [36]. The results from the three ongoing trials should provide clinicians with information about whether tranexamic acid is effective in reducing death and disability without increasing thrombotic events. The trials will also provide information about whether its effect varies by time to treatment.

**Fig. 1:**
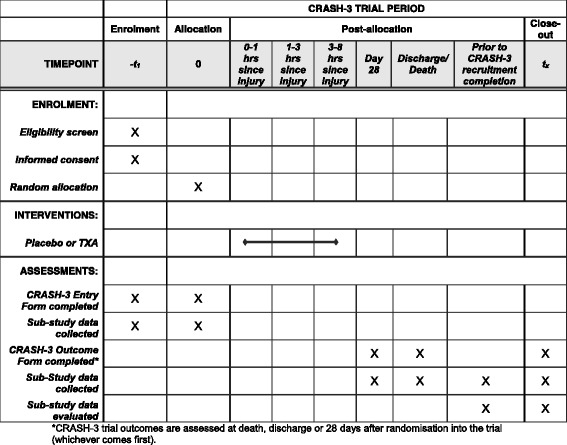
Schedule of enrolment, intervention and assessment in the CRASH-3 trial and CRASH-3 IBMS

However, these trials will not provide information about the mechanism by which tranexamic acid might exert its effects in TBI. If tranexamic acid reduces mortality by reducing intracranial haemorrhage, we would expect there to be less blood on head CT scans of tranexamic acid-treated patients, particularly those treated soon after injury [25]. If tranexamic acid increases the risk of cerebral ischaemia, we would expect to see more ischaemic lesions in tranexamic acid-treated patients, particularly in those treated after a more prolonged period following injury [37]. The CRASH-3 Intracranial Bleeding Mechanistic Sub-Study (CRASH-3 IBMS) will examine the effect of tranexamic acid on intracranial haemorrhage and cerebral ischaemia in a cohort of patients enrolled in the CRASH-3 trial. This paper outlines the protocol for the CRASH-3 IBMS and is in line with the Standard Protocol Items: Recommendations for Interventional Trials (SPIRIT) guidelines. The SPIRIT checklist and figure have been included as Additional file 1 and Fig. 1, respectively.

### Aim

The CRASH-3 IBMS aims to examine the mechanism by which tranexamic acid exerts its effects in patients with isolated TBI. Specifically, we will assess the effect of tranexamic acid on intracranial bleeding and cerebral ischaemia.

### Trial design

**Fig. 2:**
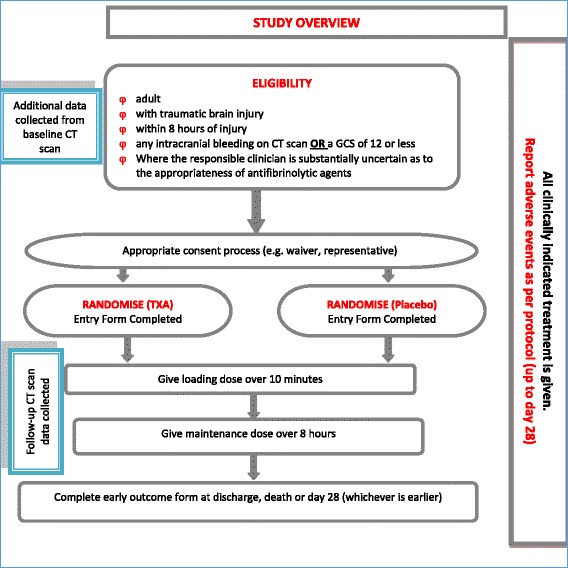
CRASH-3 trial inclusion criteria (blue boxes show additional procedure for the CRASH-3 Intracranial Bleeding Mechanistic Sub-Study)

The CRASH-3 IBMS is a mechanistic randomised controlled trial nested within a larger prospective, double-blind, multi-centre, parallel-arm, randomised, placebo controlled trial. The CRASH-3 IBMS is nested in a cohort of CRASH-3 trial participants (NCT01402882) (Fig. 2). The aims and methods of the CRASH-3 trial are presented in detail elsewhere [36].

## Methods

### Participating sub-study sites, eligibility and interventions

#### Participating hospitals

Hospitals participating in the CRASH-3 IBMS have been selected based on the number of patients enrolled into the CRASH-3 trial and the willingness of the Principal Investigator at site to take part. Four of the highest recruiting CRASH-3 trial hospital sites in the United Kingdom have been selected to take part (Royal London Hospital, London; Queen Elizabeth Hospital, Birmingham; University Hospital, Coventry; Salford Royal Hospital, Salford). Other hospitals participating in the CRASH-3 trial will be included to meet the planned sample size; these sites are to be confirmed. All regulatory and ethical approvals will be in place before the trial starts at each site.

#### Eligibility criteria

The CRASH-3 IBMS will be conducted in a cohort of approximately 1000 adult trauma patients enrolled in the CRASH-3 trial. Patients who have a Glasgow Coma Scale score of 12 or less or intracranial bleeding on a CT scan performed before randomisation into the CRASH-3 trial (i.e., a pre-randomisation CT scan), and fulfil the inclusion criteria for the CRASH-3 trial, are eligible for inclusion in the CRASH-3 IBMS [36].

#### Randomisation into the CRASH-3 trial

TBI patients eligible for inclusion into the CRASH-3 trial are randomly allocated to receive tranexamic acid or matching placebo (0.9% sodium chloride) and the trial treatment is started as soon as possible. Patients are randomised by selecting the lowest available numbered pack from a block of eight treatment packs. Randomisation codes are generated with a computer random number generator. There is no need to withhold any clinically indicated treatment in the CRASH-3 trial. Tranexamic acid or placebo is provided as an additional treatment to the usual management of TBI. The loading dose of the trial treatment is administered by intravenous injection immediately after randomisation (within minutes). The maintenance dose (by intravenous infusion) should start as soon as the loading dose is completed.

#### Adverse events in the CRASH-3 trial

Any untoward medical occurrence affecting a trial patient up to 28 days after randomisation will be reported in line with the CRASH-3 trial protocol. If the patient develops an adverse event during the treatment phase, the trial drug should be stopped. In this situation, the patient should be treated in line with local procedures and then followed up. The independent Data Monitoring Committee may recommend for the early termination of the trial, and the final decision lies with the Trial Steering Committee.

#### Unblinding before the end of the CRASH-3 trial

If there are contraindications to tranexamic acid following randomisation, the trial treatment should be stopped and all standard clinical care provided. Unblinding is only necessary if the clinician believes that clinical management depends importantly upon knowledge of whether the patient received tranexamic acid or placebo. In this case, a 24 hour telephone service is available to confirm whether the patient received tranexamic acid or placebo.

### Outcomes and outcome measurement

#### Primary outcome

The total volume of intracranial bleeding after randomisation, adjusting for total volume of intracranial bleeding at baseline if baseline volume is available.

#### Secondary outcomes

1. Frequency of progressive haemorrhage – number of patients with a post-randomisation CT scan with total haemorrhage volume of more than 25% of the volume on the pre-randomisation scan;
2. Frequency of new haemorrhage – number of patients with haemorrhage on the post-randomisation CT scan when there was not one on the pre-randomisation scan;
3. New focal ischaemic lesions – ischaemic lesions which appear on a post-randomisation scan but not on the pre-randomisation scan;
4. Total volume of intracranial bleeding after randomisation in patients who undergo surgical evacuation of haemorrhage, adjusting for volume of baseline bleeding.

All outcomes will be compared across treatment groups.

#### Outcome measurement: estimating haemorrhage volume

Patients often undergo one brain CT scan as part of routine medical care prior to randomisation into the CRASH-3 trial. The majority of patients are scanned again after randomisation into the CRASH-3 trial. In the CRASH-3 IBMS, we will measure the volume of intracranial haemorrhage on pre- and post-randomisation CT scans. A simple validated scale for measuring intracranial haemorrhage volume shows good agreement with the gold standard of computer-assisted volumetric analysis, which requires demarcation of the haemorrhage borders [38–49].

The ABC/2 method is a quick and easy technique used to estimate the volume of intracranial haemorrhage [50]. This method assumes haematoma volume is approximately equal to an ellipsoid shape (i.e., three dimensional oval shape). For ease of assessment, the formula for calculating the volume of an ellipsoid (4/3 × π × (A/2) × (B/2) × (C/2)) can be simplified to ABC/2 if we assume π is equal to 3. This method selects a representative slice near the centre of the haematoma on which the bleed is most visible. On this slice, two measurements are taken: (A) the maximal diameter; (B) width perpendicular to A. For the measurement of depth, the maximal number of slices on which the haematoma is visible is multiplied by slice thickness (C). These three measurements are multiplied and the sum divided by two (ABC/2) to provide the volume measurement in cm^3^.

Whilst some researchers have found that the ABC/2 method overestimates lesion volume compared to computer-assisted methods [39, 44, 45, 47, 51–55], others claim the opposite [41, 56]. Haemorrhagic lesions that have a regular shape are more accurately estimated using the ABC/2 method compared to lesions with irregular or multi-lobular shapes [43, 45–56]. Furthermore, a number of variations of the ABC/2 method adjust for the depth of a lesion. Whilst some have found that adjusting for depth significantly underestimates volumes because smaller slice volumes are eliminated [57], others found that adjusting for depth is favourable [48].

Although the ABC/2 method is a less specific measure of haemorrhage volume and overestimation due to false positives would dilute the effect of the treatment towards the null, its low sensitivity and underestimation due to false negatives would not impact the effect of the treatment on haemorrhage. Furthermore, the more accurate method of estimating haemorrhage would have been more expensive and therefore administered in a smaller number of patients given the limited budget of a clinical trial. Although a more accurate method in a small trial would result in less measurement error, a less accurate method in a larger trial would result in less random error. We believe that the ABC/2 method is sufficiently accurate and therefore chose to use this method in a larger trial. Furthermore, the assessor rating the scans will be blind to treatment allocation and thus the bias from measurement error should be balanced between treatment groups.

#### Total haemorrhage volume

The total haemorrhage volume on each scan will be calculated by totalling the volumes of intra-parenchymal, intra-ventricular, epidural and subdural haemorrhage.

### Estimating intra-parenchymal, intra-ventricular and epidural haemorrhage volume using ABC/2

Volume estimation of intracranial haemorrhage is aided by the characterisation of haematomas. The final shape of a haematoma is influenced by its location. Intra-axial (or intra-cerebral) haematomas include intra-parenchymal haematomas, which occur in the brain tissue, and intra-ventricular haematomas, which occur in the ventricles of the brain. These haematomas tend to have regular shapes that are clearly definable in every dimension (i.e., their length, width and depth can be measured on a CT scan). Extra-axial haematomas occur between the three membranes that surround the brain (dura mater, arachnoid mater and pia mater). Epidural haematomas are a type of extra-axial haematoma and occur between the skull and outer membrane of the central nervous system (dura mater). They have a clear shape that can be measured in every dimension. The ABC/2 method assumes the haemorrhage has an ellipsoid shape and has been validated in intra-parenchymal [38], intra-ventricular [46] and epidural haematomas [47, 48]. We will estimate the volume of intra-parenchymal, intra-ventricular and epidural/extradural haemorrhage using the ABC/2 method.

### Estimating subdural haemorrhage volume using maximum width

Subdural haematomas are another type of extra-axial haemorrhage and occur between the dura mater and the middle membrane of the central nervous system (arachnoid mater). Subdural haematomas are crescent shaped as they follow the pattern of the brain’s convexity. The exact limits of a subdural haematoma are not clearly definable in any dimension. This type of haemorrhage can theoretically occupy the entire subdural space. Given that the ABC/2 method assumes the haemorrhage has an ellipsoid shape, it would not provide an accurate volume estimation of subdural haemorrhage. Indeed, there have been reports of underestimation in subdural haemorrhage volume when using an adapted version of the ABC/2 method compared with computer-assisted volumetric analysis [41, 56].

Some researchers and clinicians propose that it is more appropriate to estimate subdural haemorrhage volume using a formula which takes the difference between two spheres (representing the entire subdural space), divides by two (as subdural haemorrhage is usually unilateral) and divide by two again (as subdural haemorrhage tends to be thicker at the centre and thinner at the sides). This method has been tested at the Neurosurgical Trauma Unit at the Queen Elizabeth Hospital in Birmingham (UK) and has been shown to provide more clinically relevant estimates of haemorrhage volume than the ABC/2 method [58]. Although this method overestimates subdural volume, it is less than the error provided by the ABC/2 method. The key measurement in determining the clinical significance of a subdural haemorrhage is its thickness (i.e., the B measurement when using the ABC/2 method) [59]. In the CRASH-3 IBMS, we will measure the maximum width of a subdural bleed, and compute its volume using the aforementioned formula.

### Measurement of subarachnoid haemorrhage

Subarachnoid bleeds are another type of extra-axial haemorrhage and occur in the area between the arachnoid membrane and the innermost membrane surrounding the brain (pia mater). The shape of the subarachnoid space resembles a spider’s web and therefore haemorrhage in the subarachnoid space cannot be clearly measured in any dimension. Although there are a number of CT grading scales that include the characterisation of subarachnoid haemorrhage [60, 61], they are criticised for being subjective and not comprehensive enough to serve as a primary grading scale for this type of haemorrhage [62]. For example, the Fisher scale and its modified version do not consider subarachnoid haemorrhage in isolation but in combination with intraventricular haemorrhage [63].

In the CRASH-3 IBMS, the size of a subarachnoid haemorrhage will be characterised as small, medium or large. Each bleed will then be described as focal (localised to a specific location), multiple (not localised but not widespread) or diffuse (widespread). This method is also subjective and may have low sensitivity and specificity, therefore misclassification would bias the effect of the treatment towards the null value. We hope that, by using this method in a large trial, the bias from measurement error would be offset by a reduction in random error.

### Petechial haemorrhage

Petechial haemorrhage manifests as a very small dot on a CT scan. CT scans and accompanying radiology reports will be examined to indicate whether petechial haemorrhage is present.

### Outcome measurement: focal ischaemic lesions

Ischaemic stroke is due to the compromise of blood and oxygen flow through either large or small arteries supplying the brain parenchyma. Thrombotic occlusion of intracranial vessels produce wedge-shaped cortical infarctions.

Cerebral ischaemia would reliably manifest on a CT scan performed at least 48 hours after randomisation [62]. However, given that clinical scans are performed for diagnostic purposes, it is not possible to carry out scans at set time points post-randomisation. Brain imaging techniques, including MRI diffusion weighted imaging, have higher sensitivity and specificity compared to CT in the early diagnosis of ischaemic infarction, and are often clinically warranted when there is a suspected stroke. Therefore, the assessor will examine all available brain scans performed within 28 days of randomisation and the accompanying radiology reports for evidence of focal ischaemic lesions and record the time from randomisation to detection.

Furthermore, given that CT imaging is the first and most common neuroimaging examination performed for emergency assessment of suspected acute haemorrhage and stroke around the world [64, 65], the majority of scans included in the CRASH-3 IBMS will be CT scans. Therefore, it is important to clarify how we will capture this endpoint when only CT scans are available. Cerebral infarction manifests as wedge-shaped low attenuation on a CT scan. Given that oedema also manifests as low attenuation on CT, the radiology reports that accompany CT scans should indicate whether the low attenuation is representative of oedema or infarction. Brain imaging reports often refer to cerebral infarction by the affected vascular territory (e.g., anterior cerebral artery, middle cerebral artery, posterior cerebral artery, lacunar, cerebellar, brainstem). The assessor will examine all available brain imaging to assess whether oedema or infarction can be excluded given the appearance of earlier scans. For example, some patients have oedematous haemorrhagic lesions, which on CT manifests as high density haemorrhage surrounded by low density oedema. In later scans the haemorrhage may resolve but the oedema may remain. If only considered alone, the later CT scan may have the appearance of infarction but could be representative of residual oedema. We will attempt to minimise such errors by comparing the appearance of cerebral infarction/oedema between consecutive scans, and consider the accompanying scan reports for radiological opinion. If the available scans and accompanying reports are unable to confirm the presence of an ischaemic lesion, we would seek further radiological and clinical opinion.

### Outcome measurement: mass effect and other CT endpoints

Space-occupying intracranial lesions can displace brain tissue. The shift of midline structures past the centre line of the brain will be measured in millimetres. We will also record whether mass effect has caused ventricular and sulcal effacement.

All scans will be rated according to the Marshall classification – the most extensively used CT classification scale in TBI [66]. Three main characteristics define the Marshall classification, namely presence of mass lesion, degree of compression of perimesencephalic cisterns and degree of midline shift.

### Sample size

Assuming the average baseline intracranial bleeding volume is 20 mL and assuming the same average increase (8 mL), standard deviation (28 mL) and correlation (rho = 0.6) between baseline and follow-up bleeding volumes as in the control group of the CRASH-2 Intracranial Bleeding Sub-study [34], a study with at least 1000 participants will have 80% power (at alpha = 0.05) to detect a 15% lower bleeding volume in the tranexamic acid group at follow-up (i.e., 24 mL tranexamic acid vs. 28 mL placebo). In the main CRASH-3 trial, we hypothesise that tranexamic acid will reduce intracranial bleeding by approximately 15%. The sample size estimates have been reviewed and approved by statisticians at the London School of Hygiene and Tropical Medicine.

### Data collection, management and analysis

#### Procedures for data collection

The CRASH-3 trial database will be used to prepare a list of all patients with a Glasgow Coma Scale score of 12 or less or with a pre-randomisation CT scan at participating sub-study hospitals. The list will include unique randomisation (box and pack) numbers, date and time of randomisation, and time between injury and randomisation into the CRASH-3 trial. The randomisation numbers will be used at the participating site to identify the patient using their hospital number. The latter will be used at the participating hospital to identify the patient. The outcome assessor (research fellow with training in brain imaging assessment) will hold a letter of access at the participating hospital and use the patient hospital number to retrieve pre- and post-randomisation scans from the hospital imaging system. The outcome assessor will complete the outcome forms at each site using the relevant scans and accompanying radiology reports. All the data are collected by the same outcome assessor who is blind to treatment allocation.

If the patient does not have a pre-randomisation scan, only the post-randomisation scan form is completed. If the patient does not have a post-randomisation scan, only the pre-randomisation scan form is completed. We record whether pre- and/or post-randomisation scans are available such that we can examine missing data by trial arm.

In most cases, the post-randomisation scan is the first scan performed after randomisation, which is normally within 72 hours of randomisation. Furthermore, due to ongoing clinical management, some patients are scanned within minutes of randomisation. Tranexamic acid would not have had sufficient opportunity to effect haemorrhage or infarction in such a way that would manifest on a scan this soon after randomisation. Therefore, for patients scanned within minutes of randomisation, we also measure all the outcomes of interest on the next available post-randomisation scan, which is normally closer to 72 hours of randomisation. All available brain imaging is examined for evidence of focal ischaemic lesions.

The time stamped on the scans will be used to calculate the following time intervals: (1) the time between injury and the pre-randomisation CT scan and (2) the time between randomisation into the trial and the post-randomisation scan. If a patient has undergone neurosurgery following their injury, information on the date and time of neurosurgery will be collected using prospective reports including patient anaesthetic charts. The outcome data is collected for all patients included in the CRASH-3 IBMS (unless consent was withdrawn) irrespective of whether the trial treatment was received (i.e., on an intention-to-treat basis). The outcome data is directly uploaded into an electronic database accessed at each sub-study site.

#### Data management plan

A data management plan will be prepared in advance of data collection (Additional file 2). This will detail all aspects of data collection and recording to ensure compliance with International Conference on Harmonisation Good Clinical Practice guidelines (ICH-GCP) [67], United Kingdom Clinical Trials Regulations and the Data Protection Act [68]. Data will be recorded in a database developed in line with relevant regulatory requirements, including ICH-GCP guidelines.

#### Statistical analysis

##### Primary outcome

A linear regression model will examine the primary outcome; whether receipt of the trial treatment can predict total haemorrhage volume following randomisation. Mean haemorrhage volume will be compared between trial arms, adjusting for baseline haemorrhage volume. Adjusting for baseline haemorrhage volume is important as it is a strong predictor of haematoma increase [17, 69, 70], meaning that the baseline adjustment can increase the power of the comparison by reducing the impact of between-patient variability. We will conduct subgroup analysis to examine whether the effect of tranexamic acid on intracranial haemorrhage is modified by time to treatment. A subgroup analysis by time is important as previous evidence suggests that the effect of tranexamic acid is strongly dependent on how quickly after injury it is received (CRASH-2).

##### Secondary outcomes

We will express the effect of tranexamic acid on the occurrence of dichotomous CT endpoints, including progressive haemorrhage or new haemorrhage, using relative risks and 95% CIs estimated using generalised linear models.

We will express the effect of tranexamic acid on new focal cerebral ischaemic lesions measured at several post-randomisation time-points using relative risks and 95% CIs estimated using generalised linear mixed models to account for the fact that this outcome could be measured at several time-points following randomisation.

##### Missing data

In line with the Consolidated Standards of Reporting Trials [71], we will report the number of patients without pre- and post-randomisation scans by treatment arm. If the outcome of interest (haemorrhage expansion) is associated with the reason the data are missing (patients with haemorrhage expansion may be more likely to die before the second scan), imbalance in missing data by treatment group can cause bias. If we suspect that data are missing not at random [72], we will conduct sensitivity analysis to explore the implications.

##### Between-centre effects

There is no evidence for the hypothesis that between-centre differences in unfavourable outcome affect the chance of demonstrating a treatment effect in randomised trials of TBI [73]. This study estimated the between-centre differences beyond the random variation that may result from some centres that only treat a small number of patients. Given this evidence and that we have no biological or mechanistic explanation to expect any variation in a treatment effect between centres, we do not anticipate to find centre effects in the CRASH-3 IBMS. Furthermore, the majority of hospitals included in the CRASH-3 IBMS are in western countries. The homogeneity in patient characteristics and care facilities is further reason not to expect a between-centre difference in treatment effect. However, for the purpose of transparency we will report the interaction between centre and treatment effect using a logistic regression model with interaction between centre and treatment.

##### Inter-rater reliability

The inter-rater reliability of haemorrhage occurrence will be assessed using relevant Entry Form data from the CRASH-3 trial to examine consistency among ratings provided by the research fellow and clinical staff.

##### Interim and final analyses

There are no interim analyses planned for the CRASH-3 IBMS. The final analysis for the CRASH-3 IBMS will be undertaken following completion of the main CRASH-3 trial. A complete statistical analysis plan will be published separately prior to completion of the CRASH-3 trial.

### Monitoring

All data for the CRASH-3 trial will be subject to statistical monitoring and approximately 10% of data will be subject to on-site monitoring. Consent forms will be monitored centrally by the Trial Coordinating Centre (where permission is given to do so). Investigators/institutions are required to provide direct access to source data/documents for trial-related monitoring, audits, ethics committee review, and regulatory inspection. All trial-related and source documents must be kept for at least 5 years after the end of the trial. As all the CRASH-3 IBMS data will be collected directly from source data, additional monitoring will not be carried out for this data.

### Potential risks

The effective radiation dose from a CT scan is about 2 mSv, which is approximately the amount received from background radiation in 8 months. Because CRASH-3 IBMS will mainly use data from CT scans undertaken as part of routine patient care, patients will not be exposed to extra radiation. There is no additional burden or risk to the patient as a result of CRASH-3 IBMS. It is standard care for all patients with TBI and associated clinical signs to have a CT scan. Follow-up CT scans are often conducted for diagnostic purposes around 24 to 72 hours after the initial scan. Steps taken to minimise the risks associated with handling personal data will be detailed in the Confidentiality section.

### Confidentiality and dissemination

#### Confidentiality

Only staff with authorised access to the scans, either as clinicians or research contract holders, will be able to retrieve and review them. Completed scan data forms will be uploaded onto a secure database. The scan data forms will contain no patient identifiable data (Additional file 3). Scans include the date and time of the scan and this information could potentially be used by anyone with access to the hospital radiology system to identify the patient. For this reason, scan data forms will only include the randomisation number, the time interval between the injury and the scan (pre-randomisation scan form), and the time interval between randomisation and the scan (post-randomisation scan form). As no personal data will be collected, the anonymity of each patient will be protected.

#### Publication

The results from this trial will be published in peer-reviewed medical journals. Dissemination of results to patients will take place via the media, trial website (crash3.lshtm.ac.uk) and relevant patient organisations. All participating sites will be credited in key publications.

## Discussion

### Potential benefit of CRASH-3 IBMS: furthers knowledge about mechanism of action of tranexamic acid in TBI

The CRASH-3 IBMS is a nested randomised trial that will reliably examine the effect of tranexamic acid on intracranial haemorrhage and cerebral ischaemia. We hope that this trial will provide information about the mechanism of action of tranexamic acid in isolated TBI. An understanding of the mechanism of action of tranexamic acid and insight into factors that might affect this mechanism, is critical in the appropriate generalisation of trial results [74]. If patients who receive tranexamic acid have less intracranial bleeding on their CT scans compared to those who receive placebo, this information, along with the results of the main CRASH-3 trial, could inform the administration of tranexamic acid in TBI. If TBI patients who receive tranexamic acid soon after injury have less haemorrhage expansion compared to those who receive tranexamic later, then time between injury and treatment is a factor relevant to the mechanism of action which, with the results of the main CRASH-3 trial, should be considered when making treatment decisions. Furthermore, if we find evidence of cerebral ischaemia in patients who receive tranexamic acid and the effect varies by time to treatment, this information can be used to prevent adverse outcomes and ensure those receiving tranexamic acid are those most likely to benefit from it. Therefore, the knowledge gained from the nested CRASH-3 IBMS will add to the evidence base and could benefit the clinical management of patients with head injuries.

Furthermore, the patients included in the CRASH-3 IBMS are likely to have more severe head injuries compared to patients in the CRASH-3 trial but not included in the CRASH-3 IBMS. The patients in the sub-study are not a random sample of patients in the CRASH-3 trial, nor will they be comparable. It is not necessary for the sub-study population to be representative of the CRASH-3 trial population because knowledge about a causal mechanism facilitates generalisation and not representativeness of the trial patients [75]. If the sub-study used a random sample of patients from the CRASH-3 trial, the results would not necessarily apply to either more or less severe patients, but only to a hypothetical patient of average injury severity. Representativeness of trial patients does not help us to generalise our findings to other TBI patients. Knowledge about whether tranexamic acid reduces intracranial bleeding or increases cerebral ischaemia will inform the administration of tranexamic acid in TBI and allow us to appropriately generalise the trial results.

### Potential dangers of CRASH-3 IBMS: power and alternative mechanisms leading to death in TBI

The CRASH-3 trial and CRASH-3 IBMS are based on the premise that intracranial haemorrhage is the mechanism that leads to death in patients with TBI. We hypothesise that tranexamic acid will reduce intracranial haemorrhage, which will in turn reduce the risk of death and disability. We assume that, by inhibiting fibrinolysis, tranexamic acid increases blood viscosity, reduces blood flow and slows the rate of haemorrhage (Poiseuille’s Law [76]). However, it is possible that tranexamic acid does reduce intracranial haemorrhage but the CRASH-3 IBMS might not have sufficient power to detect such an effect. Our sample size calculation is based on a specific difference in haemorrhage volume between treatment groups. If receiving tranexamic acid results in a smaller reduction in haemorrhage volume than we have assumed, the CRASH-3 IBMS might not detect it and we may falsely conclude that tranexamic acid does not reduce intracranial haemorrhage. This is a limitation of conducting this nested sub-study in a smaller population of the main trial population. There is a trade-off between a larger sample, which would allow us to detect a smaller treatment effect and time, and resources; therefore, we have estimated a realistic sample size based on the best available evidence in this area.

Furthermore, if tranexamic acid reduces intracranial haemorrhage in TBI patients and this is detected by the CRASH-3 IBMS, it is still possible that clinical outcomes may not improve. This could be because intracranial haemorrhage is not the mechanism that leads to death in TBI patients. It is also possible that the potential benefit of tranexamic acid in reducing intracranial haemorrhage may be offset by the increased risk of cerebral ischaemia [29, 30], particularly when administered several hours after injury when there is an increased risk of thrombotic disseminated intravascular coagulation [25]. The CRASH-3 IBMS will provide information on both endpoints and could aid the interpretation of results from the CRASH-3 trial.

## Trial status

The first patient was enrolled in the CRASH-3 trial on 20 July 2012. Recruitment is currently ongoing. It is anticipated that recruitment for the CRASH-3 trial will be complete by 31 December 2017. Data collection for the CRASH-3 IBMS started in February 2016. All data for the CRASH-3 IBMS will be collected prior to completion of the CRASH-3 trial.

## Additional files

Additional file 1: SPIRIT 2013 Checklist: Recommended items to address in a clinical trial protocol and related documents. (DOC 122 kb)

Additional file 2: Data management plan. (DOCX 726 kb)

Additional file 3: CT scan outcome forms. (DOCX 52 kb)

Additional file 4: Confirmation of funding for the CRASH-3 trial from The Moulton Charitable Foundation. (PDF 527 kb)

Additional file 5: Confirmation of funding for the CRASH-3 trial from the Joint Global Health Trials Scheme. (PDF 137 kb)

Additional file 6: Confirmation of funding for the CRASH-3 trial from the National Institute for Health Research. (PDF 82 kb)

Additional file 7: Confirmation of funding for the CRASH-3 trial from the London School of Hygiene and Tropical Medicine. (PDF 264 kb)

Additional file 8: Letter of favourable ethical opinion from the Medical Research and Ethics Committee and Health Research Authority. (PDF 106 kb)

Additional file 9: Letter of favourable ethical opinion from the Observational/Interventions Research Ethics Committee at the London School of Hygiene and Tropical Medicine. (PDF 267 kb)

## Abbreviations

CI: confidence interval
CRASH: Clinical Randomisation of an Antifibrinolytic in Significant Haemorrhage
CT: computed tomography
OR: odds ratio
RR: relative risk
SPIRIT: Standard Protocol Items: Recommendations for Interventional Trials
TBI: traumatic brain injury
