# Supplementary material for: A nested mechanistic sub-study into the effect of tranexamic acid versus placebo on intracranial haemorrhage and cerebral ischaemia in isolated traumatic brain injury: study protocol for a randomised controlled trial (CRASH-3 Trial Intracranial Bleeding Mechanistic Sub-Study [CRASH-3 IBMS])
Source: Trials. 2017 Jul 17;18:330. doi: 10.1186/s13063-017-2073-6 (PMC5513059; doi:10.1186/s13063-017-2073-6)
Supplement: Supplementary file 2 — Data management plan. (DOCX 726 kb) [file 13063_2017_2073_MOESM2_ESM.docx]

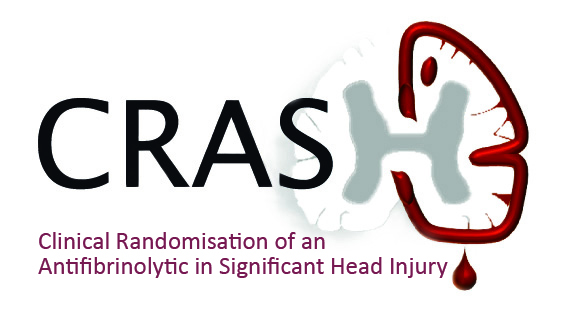
CRASH-3 Trial Intracranial Bleeding Sub-Study

Clinical Trials Unit (CTU)

London School of Hygiene & Tropical Medicine

Keppel St, London WC1E 7HT, UK

| **Working Procedure:**  **CRASH-3 INTRACRANIAL BLEEDING MECHANISTIC SUB-STUDY**  **DATA MANAGEMENT PLAN** | | |
| --- | --- | --- |
| **CURRENT VERSION SOP 1.0** | | |
| Trial title | The effect of tranexamic acid versus placebo on intracranial haemorrhage and cerebral ischaemia in isolated traumatic brain injury: a randomised trial (CRASH-3 Intracranial Bleeding Mechanistic Sub-Study [IBMS]) | |
| Current version date | Feb-2016 | |
| Review date |  | |
| Protocol code | ISRCTN15088122 | |
| Author: | PhD Student/Research Fellow:  Abda Mahmood |  |

| **Revision Chronology:** | | |
| --- | --- | --- |
| **Version Number** | **Effective Date** | **Reason for Change** |
| 1.0 | Feb-2016 | First effective version |
|  |  |  |
|  |  |  |
|  |  |  |
|  |  |  |

**WORKING PROCEDURE (WP) DODCUMENT 02:**

**DATA MANAGEMENT PLAN**

**ABBREVIATIONS**

- CRASH3-3 IBMS: CRASH-3 Trial Intracranial Bleeding Mechanistic Sub-study
- CT: Computed tomography
- CTU: Clinical Trials Unit
- DMP: Data Management Plan
- GCP: Good Clinical Practice
- ICH: International Conference on Harmonisation
- LSHTM: London School of Hygiene & Tropical Medicine
- WPs: Working Procedures

**PURPOSE**

The Data Management Plan (DMP) documents procedures that should be followed for the processing of data for the CRASH-3 Trial Intracranial Bleeding Mechanistic Sub-Study (CRASH-3 IBMS). The DMP should ensure that the integrity of the data is maintained in accord with the protocol, International Conference on Harmonisation (ICH) Good Clinical Practice (GCP)^1^ standards and applicable regulatory requirements.

**INSTITUTIONAL WP POLICY**

All Working Procedures (WPs) for CRASH-3 IBMS are produced in conjunction with London School of Hygiene & Tropical Medicine (LSHTM) policies and procedures and the WPs of the Clinical Trials Unit (CTU).

**OTHER POLICIES**

CRASH-3 IBMS will be carried out in accordance with the ICH GCP^1^ standards, national regulatory authorities’ requirements, and the CRASH-3 Trial IBMS WPs.

# BACKGROUND

The CRASH-3 IBMS is a randomised trial that examines the effect of tranexamic acid on intracranial bleeding and cerebral ischaemia in patients with isolated traumatic brain injury. This sub-study is based on a selection of approximately 1,000 patients enrolled in the CRASH-3 trial.

Many traumatic brain injury patients undergo computed tomography (CT) scanning as soon as possible after arriving in the emergency department, as part of routine medical care (i.e. before they are randomised into the CRASH-3 trial). Many patients will be scanned again for diagnostic purposes (i.e. after they are randomised into the CRASH-3 trial). This sub-study will examine pre- and post-randomisation CT scans as per the data collection forms (see Appendix 1) and methods detailed in the protocol (CRASH-3 Intracranial Bleeding Sub-Study Protocol). The Data Management Plan for the CRASH-3 IBMS will be in conjunction with the CRASH-3 trial Data Management Plan (CRASH-3 02 Data Management Plan).

Anonymized data will be captured using a web database and exported into Excel and Stata to enable it to be summarised and analysed using Excel and Stata software. In order to ensure the integrity of the data and adopt a data driven approach:

- 1. the sub-study protocol details the aims, methods and plans for statistical analyses;
  2. the protocol and statistical analysis plan will be published in peer reviewed medical journals;
  3. the Working Procedure 01 document details the procedure of data collection;
  4. each data point in the outcome form is labelled with identifiable headings and descriptions when exported.

**RESPONSIBLE PERSONNEL**

**Research Fellow/PhD Candidate roles**: The research fellow (PhD Candidate) is responsible for collecting, managing and analysing data from the CRASH-3 IBMS. The research fellow is responsible for working with: the IT manager to develop the web database; the CRASH-3 trial manager, the senior trial manager and the project director to ensure compliance with ICH GCP^1^ standards, national regulatory authorities’ requirements, and the CRASH-3 Trial IBMS WPs; hospital research staff to extract the patient data using anonymised trial information; hospital clinical staff to resolve queries regarding scan assessment; principal investigators at site to resolve data monitoring queries; the principal investigator of the CRASH-3 trial and statistical advisors for the CRASH-3 IBMS to discuss methodological problems that may arise during data collection. Following data collection and analysis, the research fellow is responsible for working with other investigators to write up and submit the results for publication in peer reviewed medical journals, and disseminate the sub-study findings using patient organisations and relevant online platforms.

**CTU IT Manager/database developer roles**: The IT manager is responsible for developing the web database for the purpose of the CRASH-3 IBMS in consultation with the research fellow and in accord with the data collection forms (see Appendix 1) and ICH GCP standards. The IT manager is responsible for working with the research fellow to respond to release requests. The IT manager and research fellow are responsible for ensuring that all changes made to the database are documented using release request and user acceptance testing forms.

**CTU CRASH-3 Trial Manager and Senior Trial Manager:** The CRASH-3 trial manager and senior trial manager are responsible for working with the research fellow to ensure all relevant regulatory and local ethical approvals are in place for the CRASH-3 IBMS, and all amendments are documented in accord with regulatory guidelines. The trial managers are responsible for working with the research fellow to ensure that the trial master file for the CRASH-3 IBMS meets all relevant regulatory requirements.

**CTU Project Director roles**: The CTU director is responsible for working with the research fellow to oversee the progress and development of the CRASH-3 IBMS.

**CTU Principal Investigator roles**: The Principal Investigator for the CRASH-3 trial is responsible for working with the research fellow to oversee the scientific progress of the CRASH-3 IBMS.

**TIMEPLAN OF TRIAL ACTIVITIES**

| Data collection starts | Feb 2016 |
| --- | --- |
| Data collection ends | ~ December 2017 |
| Database hardlock | ~ Jan 2018 |
| Start of Result Analysis | ~ Jan 2018 |
| Completion of Close out and Publication | ~ September 2018 |
| Study Archive | ~ October 2018 |

**CRF DEVELOPMENT AND PILOTING**

The case report forms (outcome forms) will be designed according to the LSHTM SOP 025 Case Report Forms and will be part of the protocol development. The outcome forms (see Appendix 1) will be piloted at the Queen Elizabeth Hospital in Birmingham as per the method detailed in the protocol (Protocol for CRASH-3 Intracranial Bleeding Sub-Study). The piloting process took place under the supervision of Clinical Research Fellow and Neurosurgical Registrar at the Queen Elizabeth Hospital in Birmingham, Mr Dave Davies.

# DATABASE DESIGN, TESTING AND VALIDATION

- Anonymised data will be recorded in a web database developed in consultation with appropriate regulatory authorities, including ICH GCP guidelines.
  - A *Risk Assessment* was performed to identify and minimize risks and hazards of using a custom developed web database.
  - A *Form Specification Matrix* was developed to define the questions, variable names, formatting rules and other rules. The database includes required fields; certain questions must be answered otherwise a form cannot be submitted and saved.
  - The web database forms were tested to ensure data could be entered in all fields and the entered data was the same as the saved data. A live version of the database was then released.
  - Any changes made to the live version of the database are logged and approved by the IT Manager and Project Director.
- Anonymised data is entered and submitted into the web database, and the forms are automatically uploaded onto the database.
- Submitted forms can be edited and previously submitted versions of the form are saved in a log with details of who edited the data and when edits were made.
- Forms cannot be deleted by the research fellow who entered the data. If the form has been entered in error or contains patient identifiable information, the IT Manager must access the form from the server and delete the erroneous or confidential data.
- After data collection is complete at each site, the data will be exported in a csv format and checked in Excel/Stata for any missing or irregular data. If necessary, the data will be amended after 1) the research fellow consults the research/clinical staff on site for clarification; 2) the research fellow reattends the relevant site to examine queries; or 3) the research fellow checks if queries can be confirmed by checking against CRASH-3 trial data.
- The unblinded data will be analysed by the research fellow in consultation with the CTU project director, principal investigator for the CRASH-3 trial, and statistical advisors for the CRASH-3 IBMS.

# DATA VALIDATION

Both manual and electronic validation will be carried out on data.

***Manual checks***

This involves visually checking CRFs once they are submitted on the database and checking data for outliers and irregularities when exported.

***Computerised validation checks***

After it is exported from the database, data is checked to confirm it is able to capture expected relationships and is not completely random. For example, we will use statistical software (Stata) to check whether patients with larger bleeds on the pre-randomisation scan are more likely to undergo neurosurgery before the post-randomisation scan.

***Data review***

Data review will be done after data collection is complete to ensure the integrity of the data. Data will be extracted using a download report facility within the database and reviewed for logical inconsistencies.

# QUALITY CONTROL AND QUALITY ASSURANCE

Quality control procedures will be built into each of the data management activities:

- CRF Design
- Clinical trial database user acceptance testing

## Quality control of CRF Design

Case report forms will be designed according to relevant Standard Operation Procedures at the CTU.

## Clinical trial database design

The Sub-Study database will be designed, developed and tested at the CTU.

***Data validation***

The CRASH-3 trial Entry Form collects data on the location of intracranial haemorrhage on CT scan which is confirmed by a trained clinician. This data will be cross-checked against the baseline data collected as part of CRASH-3 IBMS to ensure the same haemorrhage has been recorded in the CRASH-3 trial database and CRASH-3 IBMS.

# DATABASE LOCK and UNLOCK

***Hard Lock***

Hard lock will take place at the end of the trial. After the CT scan data has been collected, the database will be locked within three months. The exact date for lock will be decided by the Principal Investigator. All unresolved queries will be resolved prior to data extraction. Hard lock will adhere to the principles in CTU SOP 033 Locking, Release and Unlocking a database, in accordance with Work Procedure Database lock and unlock. The treatment allocation code file will be sent to the Principal Investigator after database hard lock has been completed. The unblinded codes will be stored in a secure folder.

***Data Extraction***

All data stored on the web database will be extracted using the download report facility. The output will be stored in the CRASH-3 IBMS folder and automatically password protected and zipped.

***Final Analysis***

As soon as possible following database lock, the Principal Investigator will unblind the data and run the statistical analysis plan using Stata. This will be cross-checked by the named statistical advisor for the Principal Investigator’s PhD.

***Unlock***

In the event that the database has to be unlocked, permission to unlock must be given by the Trial Manager. Access must be restricted to a member of the team who has remained blinded to the results (treatment allocation). The data will be extracted prior to re-lock as detailed under hard lock. The database should then be relocked and certified as such. The Principal Investigator will check the system log to confirm that the actions detailed were carried out and no other changes were made, and sign off the report.

# SECURITY

- LSHTM IT Support will be responsible for all security, backups and recovery issues.
- The CTU Database Developer will be responsible for all CTU systems security, backup and recovery.

# CONFIDENTIALITY

The CRASH-3 IBMS will only collect data relevant to the trial. No patient names or patient identifiable information will be collected. Only unique randomisation numbers will be recorded on data forms. The randomisation number and hospital ID number will only be used to establish the identity and existence of patients at the participating sites and to cross check the CRFs and CT scans associated with a patient.

Two methods of data collection are designed/managed to ensure confidentiality:

1. Direct online entry at site by the principal investigator issued with a unique username, password and PIN.
2. Pdf outcome forms are uploaded to the CRASH-3 IBMS database. Only designated CTU staff (principal investigator and IT managers) have access to the server. Uploads are automatically logged with source IP address, date and time.

## Database Authorisation

Only authorised personnel will have access to the CRASH-3 IBMS database. Access to the trial database will be gained through a password system which includes a username, password and pin.

- Granularity of access to the database will be set by the IT Manager.
- A log file of login successes/failures/attempts will be available.

## File Transfer security

All file transfers will be done through secure protocols and files will be held on secure servers.

***Access***

The data will be held securely with restricted access that is logged.

a) Data will be stored on a dedicated server by Rackspace who are ISO27001 accredited. Copies of the data forms (image files) sent as email attachments or uploaded onto the CTU secure server will also be held on a protected network drive.

b) Access to the server is only possible for authorised individuals, who have login accounts and passwords Only individuals employed by the CTU will have access and the network folders are visible only to them. External access to the LSHTM network from the internet is protected by a firewall which operates a deny-all policy so that only identified traffic to certain allowed hosts is permitted.

c) All staff have confidentiality clauses in their contracts.

## Environment

All data will be held in a secure environment (electronic).

a) The LSHTM buildings are protected by an electronic entry system and by a security guard on-site to ensure 24-hour protection.

b) Servers are held in secure data centres within the LSHTM buildings.

c) Servers are also held by Rackspace in their secure London data centre. Rackspace manage the servers patching and updating the software.

## Backup

To prevent accidental loss of data there is a network-wide backup system.

a) The LSHTM backup system involves nightly, weekly and monthly backups of the network to tape and disk. Backups are kept in a secure area with access via electronic keypad. Tapes are stored in secure fireproof safes.

b) Backup of the entire network is made on two sites separated geographically, and in the event of a major systems failure the mirror site will retain a full backup.

c) Rackspace have daily back ups going back the last two weeks. There is an encrypted daily back up of the databases sent to the LSHTM secure network drive.

**DATA STORAGE**

- A hard copy of the Trial Master File (TMF) is held in a locked filing cabinet at the CTU – keys are held securely and restricted to trial staff only. An electronic copy of the TMF is saved on the LSHTM Shared Network. All relevant folders are saved in the order they are referred to in the TMF Index.
- The database is stored on secure servers (ISO27001 accredited data centre in London, by Rackspace) and protected by privileged password protected access. Supplementary data is stored on the LSHTM Shared Network.
- Each patient’s anonymised CT scan data is saved in the secure online database under the unique patient randomisation number. Within each form, there are labels for the different questions and subsections. When the data is exported, it will be organized by pre-defined variable names as per the Database Specification.
- The excel datasheet with pre-loaded anonymised data and the time of scans has been labelled with column headings and structured so data is entered chronologically (i.e. date and time of injury, date and time of pre-randomisation scan, date and time of post-randomisation scan, data and time of neurosurgery).

**DISSEMINATION**

- In order to fulfil ethical obligations to participants and the research community and reduce publication bias, the sub-study is registered on www.clinicaltrials.gov and ISRCTN. The protocol will be published in a peer reviewed medical journal.
- The results will be published in peer reviewed medical journals. Dissemination of results to patients will take place via the media, trial website (www.crash3@lshtm.ac.uk) and relevant patient organisations. Credit in key publications will be assigned to collaborators at participating hospitals.

**REFERENCES**

1. ICH Harmonised Tripartite Guideline for Good Clinical Practice (1996), accessible at: <http://www.ich.org/fileadmin/Public_Web_Site/ICH_Products/Guidelines/Efficacy/E6_R1/Step4/E6_R1__Guideline.pdf>
