# Supplementary material for: A nested mechanistic sub-study into the effect of tranexamic acid versus placebo on intracranial haemorrhage and cerebral ischaemia in isolated traumatic brain injury: study protocol for a randomised controlled trial (CRASH-3 Trial Intracranial Bleeding Mechanistic Sub-Study [CRASH-3 IBMS])
Source: Trials. 2017 Jul 17;18:330. doi: 10.1186/s13063-017-2073-6 (PMC5513059; doi:10.1186/s13063-017-2073-6)
Supplement: Supplementary file 3 — CT scan outcome forms. (DOCX 52 kb) [file 13063_2017_2073_MOESM3_ESM.docx]

## PRE-RANDOMISATION CT SCAN FORM

| **CRASH-3 SUB-STUDY: PRE-RANDOMISATION CT SCAN FORM**  *Complete as requested and circle where appropriate, please do not leave blanks.* | | | | | | | 1. a. 🗆 first reader  b. 🗆 second reader |
| --- | --- | --- | --- | --- | --- | --- | --- |
| 2. | a.Box |  |  |  |  | 3. Time between injury and scan (hrs): ______ | |
|  | b.Pack |  |  |  | |  |  |

**4. Haemorrhagic findings**

| a. Is there any intracranial bleeding on CT scan?  *(circle one option on each line)* | | | | | | | | | | YES | | | NO | | ***If NO, go to Question 5***  ***If YES, continue*** | | | |
| --- | --- | --- | --- | --- | --- | --- | --- | --- | --- | --- | --- | --- | --- | --- | --- | --- | --- | --- |
| **Please specify type and volume of haemorrhage:** | | | | | | | | | | | | | | | | | | |
| b. Parenchymal | YES | NO | | |  | | | | | | | | | | | | | |
| bii. Code | A | B | | | C | (ABC/2)/1000 | | | HU* | | | Note | | | | | | **Codes**  L: Left  R: Right  T: Temporal  F: Frontal  P: Parietal  O: Occipital  BG: Basal Ganglia  B: Brainstem  **Abbreviations**  A: maximal diameter (mm);  B: maximal diameter perpendicular to A (mm);  C: number of slices on which haemorrhage is visible multiplied by slice thickness (mm);  HU: Hounsfield Unit  F: Focal  M: Multiple  D: Diffuse |
|  |  |  | | |  |  | | |  | | |  | | | | | |  |
|  |  |  | | |  |  | | |  | | |  | | | | | |  |
|  |  |  | | |  |  | | |  | | |  | | | | | |  |
| c. Subdural | YES | NO | | | *(measure width only*^**^*)* | | | | | | | | | | | | |  |
| cii. Code |  | B | | |  |  | | | HU | | | Note | | | | | |  |
|  |  |  | | |  |  | | |  | | |  | | | | | |  |
|  |  |  | | |  |  | | |  | | |  | | | | | |  |
|  |  |  | | |  |  | | |  | | |  | | | | | |  |
| d. Epidural | YES | NO | | |  | | | | | | | | | | | | |  |
| dii. code | A | B | | | C | (ABC/2)/1000 | | | HU | | | Note | | | | | |  |
|  |  |  | | |  |  | | |  | | |  | | | | | |  |
|  |  |  | | |  |  | | |  | | |  | | | | | |  |
|  |  |  | | |  |  | | |  | | |  | | | | | |  |
| e. Intraventricular | YES | NO | | |  | | | | | | | | | | | | |  |
| eii. code | A | B | | | C | (ABC/2)/1000 | | | HU | | | Note | | | | | |  |
|  |  |  | | |  |  | | |  | | |  | | | | | |  |
|  |  |  | | |  |  | | |  | | |  | | | | | |  |
|  |  |  | | |  |  | | |  | | |  | | | | | |  |
| f. Petechial | YES | NO | | | Note: | | | | | | | | | | | | |  |
| g. Subarachnoid | YES | NO | | | *(tick one option per line for each haemorrhage)* | | | | | | | | | | | | |  |
| gii. code | Small | | | | | Medium | | | | | Large | | | | | | HU |  |
|  | F | | M | D | | F | M | D | | | F | | | M | | D |  |  |
|  |  | |  |  | |  |  |  | | |  | | |  | |  |  |  |
|  |  | |  |  | |  |  |  | | |  | | |  | |  |  |  |
|  |  | |  |  | |  |  |  | | |  | | |  | |  |  |  |

**5. CT characteristics**

| **Marshall Classification** *(circle YES to the most severe option only)* | | |
| --- | --- | --- |
| a. Diffuse injury I (no visible pathology) | YES | NO |
| b. Diffuse injury II (cisterns present with midline shift 0-5mm; no lesion >25cm^3^) | YES | NO |
| c. Diffuse injury III (cisterns compressed/absent with midline shift 0-5mm; no lesion > 25cm^3^) | YES | NO |
| d. Diffuse injury IV (midline shift > 5mm; no lesion > 25cm^3^) | YES | NO |
| e. Evacuated mass lesion (any lesion surgically evacuated) | YES | NO |
| f. Non-evacuated mass lesion (lesion >25cm^3^; not surgically evacuated) | YES | NO |

**6. Non-haemorrhagic findings** *(circle one option on each line)*

| a. Is there any sign of acute focal ischaemic lesion? | YES | NO | Volume: | A: ___  mm | B: ___  mm | | C: ___  mm | ABC/2: ___ cm^3^ |
| --- | --- | --- | --- | --- | --- | --- | --- | --- |
| b. Are there any oedematous lesions? | YES | NO | bi. *total volume in ml* | | |  | | |

**7. Mass effect findings** *(circle one option on each line)*

| Please specify if any of the following ***mass effect*** signs are present: | | | | | | | | |
| --- | --- | --- | --- | --- | --- | --- | --- | --- |
| a. Sulcal effacement | YES | NO |  |  |  | |  |  |
| b. Ventricular effacement | YES | NO |  |  |  |  |  |  |
| c. Midline shift | YES | NO | ci. *approximate shift (mm)* | | |  | | |
| **If yes** to any of the above, is the mass effect caused by: | | | | | | | | |
| d. Haemorrhage | YES | NO |  | | | | | |
| e. Oedema | YES | NO |  |  |  |  |  |  |
| f. Both | YES | NO |  |  |  |  |  |  |

**8. Neurosurgery**

| a. Did the patient undergo neurosurgery? | YES | NO | b. Type of surgery:  _______________________  _______________________ | c. Hours between surgery and CT scan: _______ |
| --- | --- | --- | --- | --- |

**9. Details of reading**

| a. Name of the person completing the form | ___________________________________ | b. Date of reading | ____/____/____ (dd/mm/yyyy) |
| --- | --- | --- | --- |

***^*^*** *Place curser on most visually dense portion of lesion*

***^**^*** *If the width of a subdural bleed is greater than 6mm, we can assume the volume is 27cm^3^ (i.e. to aid rating the Marshall Classification)*

| Protocol code: ISRCTN15088122 Version 1.0 \| Pre-randomisation CT Scan Form |
| --- |

## POST-RANDOMISATION CT SCAN FORM

| **CRASH-3 SUB-STUDY: POST-RANDOMISATION CT SCAN FORM**  *Complete as requested and circle where appropriate, please do not leave blanks.* | | | | | | | 1. a. 🗆 first reader  b. 🗆 second reader | | |
| --- | --- | --- | --- | --- | --- | --- | --- | --- | --- |
| 2. | a.Box |  |  |  |  | 3. Time between randomisation and scan (hrs): _______ | | | |
|  | b.Pack |  |  |  | |  |  |  |  |
| Are the follow-up CT scan parameters comparable with the initial CT scan? | | | | | | | | YES | NO |

**4. Haemorrhagic findings**

| a. Is there any intracranial bleeding on CT scan?  *(circle one option on each line)* | | | | | | | | | YES | | NO | | ***If NO, go to Question 5***  ***If YES, continue*** | | |
| --- | --- | --- | --- | --- | --- | --- | --- | --- | --- | --- | --- | --- | --- | --- | --- |
| **Please specify type and volume of haemorrhage:** | | | | | | | | | | | | | | | |
| b. Parenchymal | YES | NO | | |  | | | | | | | | | | |
| bii. Code | A | B | | | C | (ABC/2)/1000) | | | HU***** | | Note | | | | **Codes**  L: Left  R: Right  T: Temporal  F: Frontal  P: Parietal  O: Occipital  BG: Basal Ganglia  B: Brainstem  **Abbreviations**  A: maximal diameter (mm);  B: maximal diameter perpendicular to A (mm);  C: number of slices on which haemorrhage is visible multiplied by slice thickness (mm);  HU: Hounsfield Unit  F: Focal  M: Multiple  D: Diffuse |
|  |  |  | | |  |  | | |  | |  | | | |  |
|  |  |  | | |  |  | | |  | |  | | | |  |
|  |  |  | | |  |  | | |  | |  | | | |  |
| c. Subdural | YES | NO | | | *(measure width only***^**^***)* | | | | | | | | | |  |
| cii. Code |  | B | | |  |  | | | HU | | Note | | | |  |
|  |  |  | | |  |  | | |  | |  | | | |  |
|  |  |  | | |  |  | | |  | |  | | | |  |
|  |  |  | | |  |  | | |  | |  | | | |  |
| d. Epidural | YES | NO | | |  | | | | | | | | | |  |
| dii. code | A | B | | | C | (ABC/2)/1000 | | | HU | | Note | | | |  |
|  |  |  | | |  |  | | |  | |  | | | |  |
|  |  |  | | |  |  | | |  | |  | | | |  |
|  |  |  | | |  |  | | |  | |  | | | |  |
| e. Intraventricular | YES | NO | | |  | | | | | | | | | |  |
| eii. code | A | B | | | C | (ABC/2)/1000 | | | HU | | Note | | | |  |
|  |  |  | | |  |  | | |  | |  | | | |  |
|  |  |  | | |  |  | | |  | |  | | | |  |
|  |  |  | | |  |  | | |  | |  | | | |  |
| f. Petechial | YES | NO | | | Note: | | | | | | | | | |  |
| g. Subarachnoid | YES | NO | | | *(tick one option per line for each haemorrhage)* | | | | | | | | | |  |
| gii. code | Small | | | | | Medium | | | | Large | | | | HU |  |
|  | F | | M | D | | F | M | D | | F | | M | D |  |  |
|  |  | |  |  | |  |  |  | |  | |  |  |  |  |
|  |  | |  |  | |  |  |  | |  | |  |  |  |  |
|  |  | |  |  | |  |  |  | |  | |  |  |  |  |
| h. Is this a new haemorrhage (not seen on pre-randomisation scan)? | | | | | | | | | | | | | YES | NO |  |
| hi. Give details (e.g. subdural present on pre-randomisation but not follow up): | | | | | | | | | | | | | | |  |
|  | | | | | | | | | | | | | | |  |

**5. CT characteristics**

| **Marshall Classification** *(circle YES to the most severe option only)* | | |
| --- | --- | --- |
| a. Diffuse injury I (no visible pathology) | YES | NO |
| b. Diffuse injury II (cisterns present with midline shift 0-5mm; no lesion >25cm^3^) | YES | NO |
| c. Diffuse injury III (cisterns compressed/absent with midline shift 0-5mm; no lesion > 25cm^3^) | YES | NO |
| d. Diffuse injury IV (midline shift > 5mm; no lesion > 25cm^3^) | YES | NO |
| e. Evacuated mass lesion (any lesion surgically evacuated) | YES | NO |
| f. Non-evacuated mass lesion (lesion >25cm^3^; not surgically evacuated) | YES | NO |

**6. Non-haemorrhagic findings** *(circle one option on each line)*

| a. Is there any sign of acute focal ischaemic lesion? | YES | NO | Volume: | A: ___  mm | B: ___  mm | C: ___  mm | ABC/2: ____ cm^3^ |
| --- | --- | --- | --- | --- | --- | --- | --- |
| ai. Is this a new acute focal ischaemic lesion (not seen on the pre-randomisation scan)? | YES | NO |  | | | | |
| b. Are there any oedematous lesions? | YES | NO |  | | | | |

**7. Mass effect findings** *(circle one option on each line)*

| Please specify if any of the following ***mass effect*** signs are present: | | | | |
| --- | --- | --- | --- | --- |
| a. Sulcal effacement | YES | NO |  | |
| b. Ventricular effacement | YES | NO |  |  |
| c. Midline shift | YES | NO | ci. *approximate shift in mm* |  |
| **If yes** to any of the above, is the mass effect caused by: | | | | |
| d. Haemorrhage | YES | NO |  | |
| e. Oedema | YES | NO |  |  |
| f. Both | YES | NO |  |  |

**8. Neurosurgery**

| a. Did the patient undergo neurosurgery? | YES | NO | b. Type of surgery:  _______________________  _______________________ | c. Hours between surgery and CT scan: _______ |
| --- | --- | --- | --- | --- |

**9. Details of reading**

| a. Name of the person completing the form | ____________________________________ | c. Date of reading | ____/____/____ (dd/mm/yyyy) |
| --- | --- | --- | --- |

***^*^*** *Place curser on most visually dense portion of lesion*

***^**^*** *If the width of a subdural bleed is greater than 6mm, we can assume the volume is 27cm^3^ (i.e. to aid rating the Marshall Classification)*

| Protocol code: ISRCTN15088122 Version 1.0 \| Post-randomisation CT Scan Form |
| --- |
