# Supplementary material for: A nested mechanistic sub-study into the effect of tranexamic acid versus placebo on intracranial haemorrhage and cerebral ischaemia in isolated traumatic brain injury: study protocol for a randomised controlled trial (CRASH-3 Trial Intracranial Bleeding Mechanistic Sub-Study [CRASH-3 IBMS])
Source: Trials. 2017 Jul 17;18:330. doi: 10.1186/s13063-017-2073-6 (PMC5513059; doi:10.1186/s13063-017-2073-6)
Supplement: Supplementary file 5 — Confirmation of funding for the CRASH-3 trial from the Joint Global Health Trials Scheme. (PDF 137 kb) [file 13063_2017_2073_MOESM5_ESM.pdf]

Medical Research Council
2nd Floor David Phillips Building, Polaris House, North Star Avenue, Swindon,
United Kingdom SN2 1ET
Telephone +44 (0) 1793 416200
Web http://www.mrc.ac.uk/

COMPLIANCE WITH THE DATA PROTECTION ACT 1998

In accordance with the Data Protection Act 1998, the personal data provided on this form will be processed by MRC, and may be held on computerised database and/or manual files. Further details may be found in the **guidance notes**

OFFER ACCEPTANCE

Document Status: With Submitter
MRC Reference: MR/M009211/1

Offer Acceptance Details

Grant Reference:	MR/M009211/1
Grant Holder:	Professor Ian Gray Roberts
Grant Title:	The CRASH-3 Trial: Tranexamic acid for the treatment of significant traumatic brain injury.
Organisation:	London Sch of Hygiene and Trop Medicine
RO Proposal Reference:	MR/L020890/1
Announced Start Date:	1 November 2014
Announced End Date:	31 July 2018
Earliest Start Date:	1 November 2014
Latest Start Date:	1 February 2015
Document Name:	Offer Acceptance
Document Description:	Re-announcement
Document Expiry Date:	27 November 2014

Reissue Details

Date of Reissue	13 November 2014
Reason for Reissue	grant offer

Combined Funding Streams

Grant Reference	: MR/M009211/1	Start Date	:	01 Nov 2014
Organisation	: London Sch of Hygiene and Trop Medicine	End Date	:	31 Jul 2018
Organisation Reference	: MR/L020890/1	Department	:	Epidemiology and Population Health
Grant Holder	: Professor Ian Gray Roberts	Grant Status	:	Authorised
Grant Title	: The CRASH-3 Trial: Tranexamic acid for the treatment of significant traumatic brain injury.	Funding Stream	:	Combined Funding Streams

Issue Date: 13 Nov 2014 Issue Reason: Grant Offer

	Directly Incurred			Directly Allocated		Indirect Costs	Exceptions			
**Description**	**Staff**	**Travel and Subsistence**	**Other Costs**	**Investigators**	**Estate Costs**	**Indirect Costs**	**Staff**	**Other Costs**	**Payment Total**	**Status**
2014/15 Q3	£36,781.89	£10,941.24	£13,413.67	£5,261.79	£1,982.21	£16,408.58	£2,661.39	£54,827.33	£142,278.10	Due
2014/15 Q4	£36,781.89	£10,941.24	£13,413.67	£5,261.79	£1,982.21	£16,408.58	£2,661.39	£54,827.33	£142,278.10	Due
2015/16 Q1	£36,781.89	£10,941.24	£13,413.67	£5,261.79	£1,982.21	£16,408.58	£2,661.39	£54,827.33	£142,278.10	Due
2015/16 Q2	£36,781.89	£10,941.24	£13,413.67	£5,261.79	£1,982.21	£16,408.58	£2,661.39	£54,827.33	£142,278.10	Due
2015/16 Q3	£37,079.81	£11,029.87	£13,522.32	£5,304.42	£1,998.26	£16,541.49	£2,682.95	£55,271.43	£143,430.55	Due
2015/16 Q4	£37,079.81	£11,029.87	£13,522.32	£5,304.42	£1,998.26	£16,541.49	£2,682.95	£55,271.43	£143,430.55	Due
2016/17 Q1	£37,079.81	£11,029.87	£13,522.32	£5,304.42	£1,998.26	£16,541.49	£2,682.95	£55,271.43	£143,430.55	Due
2016/17 Q2	£37,079.81	£11,029.87	£13,522.32	£5,304.42	£1,998.26	£16,541.49	£2,682.95	£55,271.43	£143,430.55	Due
2016/17 Q3	£37,380.19	£11,119.21	£13,631.85	£5,347.39	£2,014.45	£16,675.48	£2,704.68	£55,719.13	£144,592.38	Due
2016/17 Q4	£37,380.19	£11,119.21	£13,631.85	£5,347.39	£2,014.45	£16,675.48	£2,704.68	£55,719.13	£144,592.38	Due
2017/18 Q1	£37,380.19	£11,119.21	£13,631.85	£5,347.39	£2,014.45	£16,675.48	£2,704.68	£55,719.13	£144,592.38	Due
2017/18 Q2	£37,380.19	£11,119.21	£13,631.85	£5,347.39	£2,014.45	£16,675.48	£2,704.68	£55,719.13	£144,592.38	Due
2017/18 Q3	£37,682.94	£11,209.27	£13,742.27	£5,390.70	£2,030.77	£16,810.55	£2,726.59	£56,170.46	£145,763.55	Due
2017/18 Q4	£37,682.94	£11,209.27	£13,742.27	£5,390.70	£2,030.77	£16,810.55	£2,726.59	£56,170.46	£145,763.55	Due
2018/19 Q1	£37,682.83	£11,209.33	£13,742.28	£5,390.64	£2,030.75	£16,810.62	£2,726.59	£56,170.46	£145,763.50	Due

Paid to Date:	£0.00
Outstanding:	£2,158,494.72
SubTotal:	£2,158,494.72
Grand Total:	£2,158,494.72

Organisation: London Sch of Hygiene and Trop Medicine

Grant Holder: Professor Ian Roberts

Grant Title: The CRASH-3 Trial: Tranexamic acid for the treatment of significant traumatic brain injury.

Starts: 1 November 2014 **Ends:** 31 July 2018 **Duration:** 45

GRANT VALUE

Funds Awarded

	Authorised FEC (£)			RC Contribution (£)			
	net	Indexation	Total	net	Indexation	Total	% FEC
DI - Staff	739,588	14,488	754,076	547,295	10,721	558,016	74
DI - T&S	220,000	4,310	224,310	162,800	3,189	165,989	74
DI - Other Costs	269,714	5,284	274,998	199,588	3,910	203,498	74
DA - Investigators	105,801	2,073	107,874	78,293	1,534	79,826	74
DA - Estate Costs	39,857	781	40,638	29,494	578	30,072	74
Indirect - Indirect Costs	329,934	6,463	336,397	244,151	4,783	248,934	74
Exception - Staff	39,600	776	40,376	39,600	776	40,376	100
Exception - Other Costs	815,802	15,981	831,783	815,802	15,981	831,783	100
**Total Value of Award**	**2,560,296**	**50,154**	**2,610,450**	**2,117,024**	**41,471**	**2,158,495**	

Cost of Access to Facilities 0
(Funds not awarded to Grant Holding Organisation)

STAFF

Staff Summary

	Authorised FEC net	RC Contribution net	Number Of Staff Months
Investigator	165,701	132,915	32
Other	558,336	413,169	274
Researcher	160,952	119,104	50

Staff and DI Investigator Details

Start Date	End Date	Duration	FTE Percent	Name or Post Identifier	Summary Fund Heading	Authorised Cost (Excluding Indexation)
1 November 2014	31 July 2018	45	1	Mr A Belli	Directly Incurred	6660.00
1 November 2014	31 July 2018	45	8	Professor R CHAUDHRI	Exception	15840.00
1 November 2014	31 July 2018	45	10	Dr PJ Edwards	Directly Incurred	30610.00
1 November 2014	31 July 2018	45	5	Dr A Fawole	Exception	9900.00
1 November 2014	31 July 2018	45	1	Professor T R E Harris	Directly Incurred	5896.00
1 November 2014	31 July 2018	45	2	Professor R Jooma	Exception	3960.00
1 November 2014	31 July 2018	45	5	Ms K Ker	Directly Incurred	7744.00
1 November 2014	31 July 2018	45	5	Professor MT Shokunbi	Exception	9900.00
