# Supplementary material for: A nested mechanistic sub-study into the effect of tranexamic acid versus placebo on intracranial haemorrhage and cerebral ischaemia in isolated traumatic brain injury: study protocol for a randomised controlled trial (CRASH-3 Trial Intracranial Bleeding Mechanistic Sub-Study [CRASH-3 IBMS])
Source: Trials. 2017 Jul 17;18:330. doi: 10.1186/s13063-017-2073-6 (PMC5513059; doi:10.1186/s13063-017-2073-6)
Supplement: Supplementary file 7 — Confirmation of funding for the CRASH-3 trial from the London School of Hygiene and Tropical Medicine. (PDF 264 kb) [file 13063_2017_2073_MOESM7_ESM.pdf]

London School of Hygiene & Tropical Medicine
(University of London)
Clinical Trials Unit
Room 180, Keppel Street, London WC1E 7HT
Tel: +44(0)20 7299 4684 | Fax: +44(0)20 7299 4663
Email: ctu@Lshtm.ac.uk

CLINICAL TRIALS UNIT

LONDON SCHOOL of HYGIENE &TROPICAL MEDICINE

20 December 2016

To whom it may concern,

I am writing to confirm that the London School of Hygiene and Tropical Medicine is partly funding the CRASH-3 study and is fully funding the CRASH-3 sub-study–

Name of trial:	CRASH-3
Name of funder:	London School of Hygiene & Tropical Medicine
Grant reference:	EPNPBH61
Funding amount:	£433,276

Name of trial:	CRASH-3 sub-study
Name of funder:	London School of Hygiene & Tropical Medicine
Grant reference:	EPAA6020
Funding amount:	£65,000

Yours sincerely,

Courtenay Howe

Courtenay Howe
Clinical Trials Unit Administrator
