# Supplementary material for: A nested mechanistic sub-study into the effect of tranexamic acid versus placebo on intracranial haemorrhage and cerebral ischaemia in isolated traumatic brain injury: study protocol for a randomised controlled trial (CRASH-3 Trial Intracranial Bleeding Mechanistic Sub-Study [CRASH-3 IBMS])
Source: Trials. 2017 Jul 17;18:330. doi: 10.1186/s13063-017-2073-6 (PMC5513059; doi:10.1186/s13063-017-2073-6)
Supplement: Supplementary file 8 — Letter of favourable ethical opinion from the Medical Research and Ethics Committee and Health Research Authority. (PDF 106 kb) [file 13063_2017_2073_MOESM8_ESM.pdf]

Health Research Authority

East of England - Essex Research Ethics Committee

The Old Chapel
Royal Standard Place
Nottingham
NG1 6FS

08 June 2016

Ms Patricia Henley
Clinical Trials Unit
London School of Hygiene and Tropical Medicine
Keppel St, London
WC1E 7HT

Dear Ms Henley

**Study title:**	**Tranexamic Acid for the treatment of significant traumatic brain injury: an international, randomised, double blind, placebo controlled trial.**
**REC reference:**	**12/EE/0274**
**Protocol number:**	**ISRCTN15088122**
**EudraCT number:**	**2011-003669-14**
**Amendment number:**	**28**
**Amendment date:**	**28 April 2016**
**IRAS project ID:**	**88262**

The above amendment was reviewed between 30 May and 08 June 2016 by the Sub-Committee in correspondence.

Ethical opinion

The members of the Committee taking part in the review gave a favourable ethical opinion of the amendment on the basis described in the notice of amendment form and supporting documentation.

The sub-committee discussed the CT scan and agreed as it is a retrospective data there is no additional exposure to participants. The sub-committee discussed if this need incorporating the Participant Information Sheet and Informed Consent Form to extract and have access to participants' medical records but agreed as the PhD student has an honorary contract and she is extracting existing data in anonymised form it is not required.

The sub-committee discussed the student addition as not listed as an investigator and where this fits in with the protocol given the background of the student (psychology with a PhD in epidemiology). The sub-committee noted the student's CV states she has received training in functional and structural brain imagery analysis and in GCP and is adequately qualified to carry out this sub-study.

The sub-committee agreed the amendment presented no ethical issues.

Approved documents

The documents reviewed and approved at the meeting were:

Document	*Version*	*Date*
Covering letter on headed paper [0_Cover letter_FINAL]		16 May 2016
Notice of Substantial Amendment (CTIMP) [1 NOSA_FINAL_signed]	28	28 April 2016
Other [2 CRASH-3_Revised REC Form_highlighted_signed_PART FORM]		13 May 2016

Other [CRASH-3_Revised REC Form_signed]		13 May 2016
Other [CV_Abda Mahmood]		
Other [CV_Haleema Shakur]		
Research protocol or project proposal [C3 CTscan_Protocol version FINAL]	1	16 March 2016

Membership of the Committee

The members of the Committee who took part in the review are listed on the attached sheet.

R&D approval

All investigators and research collaborators in the NHS should notify the R&D office for the relevant NHS care organisation of this amendment and check whether it affects R&D approval of the research.

Statement of compliance

This Committee is recognised by the United Kingdom Ethics Committee Authority under the Medicines for Human Use (Clinical Trials) Regulations 2004, and is authorised to carry out the ethical review of clinical trials of investigational medicinal products.

The Committee is fully compliant with the Regulations as they relate to ethics committees and the conditions and principles of good clinical practice.

The Committee is constituted in accordance with the Governance Arrangements for Research Ethics Committees and complies fully with the Standard Operating Procedures for Research Ethics Committees in the UK.

We are pleased to welcome researchers and R & D staff at our NRES committee members' training days – see details at http://www.hra.nhs.uk/hra-training/

**12/EE/0274:**	**Please quote this number on all correspondence**

Yours sincerely

pp Adam Garrett

Dr Gerry Kamstra
Chair

E-mail: NRESCommittee.EastofEngland-Essex@nhs.net

Enclosures: *List of names and professions of members who took part in the review*

Copy to: *Mr Gerry Leonard, Barts & The London NHS Trust*
Ian Roberts, London School of Hygiene and Tropical Medicine

East of England - Essex Research Ethics Committee

Attendance at Sub-Committee of the REC meeting on 30 May 2016

Committee Members:

Name	*Profession*	*Present*
Dr Shahira Amr	Pharmacist	Yes
Dr Gerry Kamstra (Chair)	Retired Solicitor	Yes

Also in attendance:

Name	*Position (or reason for attending)*
Mr Adam Garretty	REC Assistant
