# Supplementary material for: A nested mechanistic sub-study into the effect of tranexamic acid versus placebo on intracranial haemorrhage and cerebral ischaemia in isolated traumatic brain injury: study protocol for a randomised controlled trial (CRASH-3 Trial Intracranial Bleeding Mechanistic Sub-Study [CRASH-3 IBMS])
Source: Trials. 2017 Jul 17;18:330. doi: 10.1186/s13063-017-2073-6 (PMC5513059; doi:10.1186/s13063-017-2073-6)
Supplement: Supplementary file 9 — Letter of favourable ethical opinion from the Observational/Interventions Research Ethics Committee at the London School of Hygiene and Tropical Medicine. (PDF 267 kb) [file 13063_2017_2073_MOESM9_ESM.pdf]

London School of Hygiene & Tropical Medicine
Keppel Street, London WC1E 7HT
United Kingdom
Switchboard: +44 (0)20 7636 8636
www.lshtm.ac.uk

Observational / Interventions Research Ethics Committee

Miss Danielle Beaumont
LSHTM

24 May 2016

Dear Danielle

Study Title: CRASH-3 Trial: CT Scan Sub study

LSHTM Ethics Ref: 11535

Thank you for your application for the above research project which has now been considered by the Interventions Committee via Chair's Action.

Confirmation of ethical opinion

On behalf of the Committee, I am pleased to confirm a favourable ethical opinion for the above research on the basis described in the application form, protocol and supporting documentation, subject to the conditions specified below.

Conditions of the favourable opinion

Approval is dependent on local ethical approval having been received, where relevant.

Approved documents

The final list of documents reviewed and approved is as follows:

Document Type	File Name	Date	Version
Sponsor Letter	LSHTM sponsorship confirmation 21oct11	21/10/2011	1.0
Other	DMC charter final 0.2_14 Jun12	14/06/2012	0.2
Information Sheet	Information sheet for patient & representative UKVersion 1.1	19/07/2012	1.1
Information Sheet	Consent form for patient & representative UK Version 1.1	19/07/2012	1.1
Local Approval	12-EE-0274 favourable opinion_APPROVAL	19/07/2012	1.0
Safety Information	02 SmPC updated 29 November 2012	29/11/2012	1.0
Protocol / Proposal	C3 CTscan_Protocol version FINAL	16/03/2016	1.0
Investigator CV	CV_Abda Mahmood	25/04/2016	Final
Investigator CV	CV_Ian Roberts	25/04/2016	Final
Investigator CV	CV_Haleema Shakur	25/04/2016	Final
Covering Letter	1 Ethics Submission Response_FINAL	17/05/2016	1.0

After ethical review

The Chief Investigator (CI) or delegate is responsible for informing the ethics committee of any subsequent changes to the application. These must be submitted to the committee for review using an Amendment form. Amendments must not be initiated before receipt of written favourable opinion from the committee.

The CI or delegate is also required to notify the ethics committee of any protocol violations and/or Suspected Unexpected Serious Adverse Reactions (SUSARs) which occur during the project by submitting a Serious Adverse Event form.

An annual report should be submitted to the committee using an Annual Report form on the anniversary of the approval of the study during the lifetime of the study.

At the end of the study, the CI or delegate must notify the committee using the End of Study form.

All aforementioned forms are available on the ethics online applications website and can only be submitted to the committee via the website at: http://leo.lshtm.ac.uk.

Further information is available at: www.lshtm.ac.uk/ethics.

Yours sincerely,

Professor John DH Porter
Chair

ethics@lshtm.ac.uk
http://www.lshtm.ac.uk/ethics/
