# Supplementary material for: A nested mechanistic sub-study into the effect of tranexamic acid versus placebo on intracranial haemorrhage and cerebral ischaemia in isolated traumatic brain injury: study protocol for a randomised controlled trial (CRASH-3 Trial Intracranial Bleeding Mechanistic Sub-Study [CRASH-3 IBMS])
Source: Trials. 2017 Jul 17;18:330. doi: 10.1186/s13063-017-2073-6 (PMC5513059; doi:10.1186/s13063-017-2073-6)
Supplement: Supplementary file 4 — Confirmation of funding for the CRASH-3 trial from The Moulton Charitable Foundation. (PDF 527 kb) [file 13063_2017_2073_MOESM4_ESM.pdf]

The Moulton Charitable Foundation
The Mount
Church Street
Shoreham
Sevenoaks
Kent
TN14 7SD
Tel: 01959 524008
Fax: 01959 525809
e-mail: jon.moulton@btopenworld.com

RECEIVED
31 JAN 2011

27th January 2011

Dr Pablo Perel
London School of Hygiene & Tropical Medicine
Keppel Street
London
WC1E 7HT

Dear Dr Perel,

CRASH 3 Efficacy Trial (Clinical Randomisation of an Antifibrinolytic in Significant Head Injury)

I am delighted to be able to inform you that the Trustees have selected your project for financing. Well done!

The finance of £433,276 is subject to the following:-

- Receipt and approval of your final budget with calendarisation of expenditure.
- Finance is expected to be disbursed in two to four instalments. Please provide detailed progress milestones to be reached for each instalment and dates when payments should be made.
- Funds will only be payable on receipt of an invoice to The Moulton Charitable Foundation together with a progress report confirming that the relevant milestones have been met for that instalment.
- Progress reports are required every quarter (i.e. 31st March, 30th June, 30th September & 31st December each year). Again, no payments will be made if quarterly reports are overdue.
- Confirmation in writing to the Trustees that:-

- All findings from your trials will be made available for publication and efforts will be made for publication.
- No intellectual property generated from the trial will be retained for commercial exploitation.
- That within 30 days of the end of each calendar quarter the Trustees will receive a full progress report.
- At completion of the trial a presentation of the results will be made (probably in London) to the Trustees.

Subject to the above all being finalized, funding is rapidly available.

Yours sincerely,

Jon Moulton
Trustee

Registered Name: J P Moulton Charitable Foundation
Registered Office: The Mount, Church Street, Shoreham, Sevenoaks Kent TN14 7SD.
Place of Registration: UK
Company Number: 5311748

London School of Hygiene & Tropical Medicine

(University of London)

Clinical Trials Unit

Pablo Perel
LSHTM, Room 182, Keppel Street, London WC1E 7HT
Tel: +44(0)20 7958 8120 Fax: +44(0)20 7299 4663
Email: pablo.perel@Lshtm.ac.uk

contributing to health worldwide

2 February 2011

J P Moulton Charitable Foundation
The Mount, Church Street
Shoreham, Sevenoaks
TN14 7SD

Dear Mr Moulton,

We were delighted to hear the good news that J P Moulton Charitable Foundation has selected the CRASH-3 trial for financing and we look forward to starting this important trial as soon as possible. We hope that the payments could be made in two installments and that the first installment would allow us to start on 1 March 2011.

As requested, we are attaching:

- the final budget with calendarisation of expenditure, and
- details of the progress milestones to be reached for each installment

We confirm that in line with our publication policy, all findings from the CRASH-3 trial will be made available for publication. In addition, we plan to make the data available freely to the public to facilitate further research soon after publication.

We anticipate that no new intellectual property will be generated from the trial as the trial treatment to be investigated has already passed its patent period. In addition, we encourage free use of our trial methodology.

We will be pleased to present full quarterly reports to the Trustees in the format we presented for the CRASH-2 trial and look forward also to sharing the final results with the Trustees in due course.

We thank you for continuing to support our programme of work in trauma care research. Please let me know if you require any further information.

Yours sincerely,

Pablo Perel MD, MSc, PhD
Clinical Lecturer
