# Supplementary material for: A nested mechanistic sub-study into the effect of tranexamic acid versus placebo on intracranial haemorrhage and cerebral ischaemia in isolated traumatic brain injury: study protocol for a randomised controlled trial (CRASH-3 Trial Intracranial Bleeding Mechanistic Sub-Study [CRASH-3 IBMS])
Source: Trials. 2017 Jul 17;18:330. doi: 10.1186/s13063-017-2073-6 (PMC5513059; doi:10.1186/s13063-017-2073-6)
Supplement: Supplementary file 6 — Confirmation of funding for the CRASH-3 trial from the National Institute for Health Research. (PDF 82 kb) [file 13063_2017_2073_MOESM6_ESM.pdf]

Evaluation, Trials and Studies Coordinating Centre
University of Southampton
Alpha House
Enterprise Road
Southampton
SO16 7NS

Tel: 023 8059 5586
Fax: 023 8059 5639
Email: info@netscc.ac.uk
Web: www.nets.nihr.ac.uk

Professor Ian Roberts
CTU Director
Professor of Epidemiology and Public Health
London School of Hygiene & Tropical Medicine
Epidemiology and Population Health
Keppel Street
London
WC1E 7HT

9 October 2014

Dear Professor Ian Roberts,

HTA Project: 14/190/01 - The CRASH-3 Trial: Tranexamic acid for the treatment of significant traumatic brain injury

I am pleased to inform you that your above-titled proposal has been recommended for funding. Please would you inform your co-applicants of the decision.

You will shortly receive a task to provide sponsor signatures ready for the studies contract. Please log into your user account for the NETSCC Management Information System (NETSCC MIS) at https://netscc-mis.nihr.ac.uk to complete the 'Provide Final App Signatures' task. You will need to obtain final signatures to confirm that your supporting roles agree to any changes and support your application. Please note that co-applicants are not required to sign.

Please can you also email a clean copy of your final full project description as a pdf to htafwc@soton.ac.uk.

A member of the HTA Programme, Monitoring team will be contacting you soon to request key project information and to facilitate the timely start-up of your study.

Please note that this award is not secure until the work has been contracted. Therefore you must not publicise this award until you receive a completed contract from us.

After the contracts have been signed, the HTA Programme encourages successful applicants to consider submitting their protocols for review to journals.

Congratulations once again and we wish you all success with your project.

If you have any queries, please contact me on htafwc@soton.ac.uk. I look forward to receiving your response.

Yours sincerely,

Zoe Hurworth
The HTA Programme
cc Mrs Kim Wherry, Finance and Contracts Manager, NETSCC
